# Flow velocity sensors arrangement for vegetated channels

**DOI:** 10.3389/fpls.2022.960103

**Published:** 2022-08-11

**Authors:** Yi Zhou, Weiwei Yao, Xiangli He, Tongshu Li, Shiyu Wang, Yu Han

**Affiliations:** ^1^College of Water Resources and Civil Engineering, China Agricultural University, Beijing, China; ^2^State Key Laboratory of Hydraulics and Mountain River Engineering, Sichuan University, Chengdu, China

**Keywords:** sensors arrangement, ultrasonic sensor, vegetated channel, open channel, plant protection

## Abstract

Ecological rivers or ecological channels are being widely used. Precious measurement and estimation of flow in irrigation areas are important issues in agricultural engineering. For the sustainable development of vegetation, it is necessary to consider how to use sensors to measure flow more easily in the river to protect both plants and sensors from damage. This article selects smooth channels and ecological channels of different shapes for research and presents a simplified method for arming ultrasonic sensors to obtain channel flow velocity. The flow characteristics along the normal line direction are obtained by theoretical analysis. The method uses the average flow velocity based on the normal to the channel wall to determine the location of the sensors. It combines the flow velocity determined by the sensors with the flow calculation method, thus simplifying the flow estimation steps. Experiments under flow conditions validate the efficacy of the proposed ultrasonic sensor arrangement method. This article not only simplifies the arrangement of sensors in channel flow but also improves the accuracy of the flow measurement method, which is important to promote the construction of ecological channels.

## Introduction

A key focus of the efficient and safe operation of smart irrigation districts is the measurement and monitoring of fluid flow (Gupta et al., 2021). Ecological flow is an important monitoring data for ecological channels. Riparian vegetation is particularly sensitive to fluctuations in water flow and level (Lozanovska et al., 2020). Additionally, the presence of vegetation can cause some disturbance to the flow field and flow measurement, and the presence of vegetation patches significantly altered the characteristics of wind-caused waves, water flow, and material transport in the shallow lake (Wang et al., 2021; Wu et al., 2022). At present, for flow measurement, most of them use manual handheld methods, which are more difficult to measure. Therefore, it is necessary to introduce a sensor device and study a simplified method of sensor placement in ecological/non-ecological channels to obtain flow.

**FIGURE 1:**
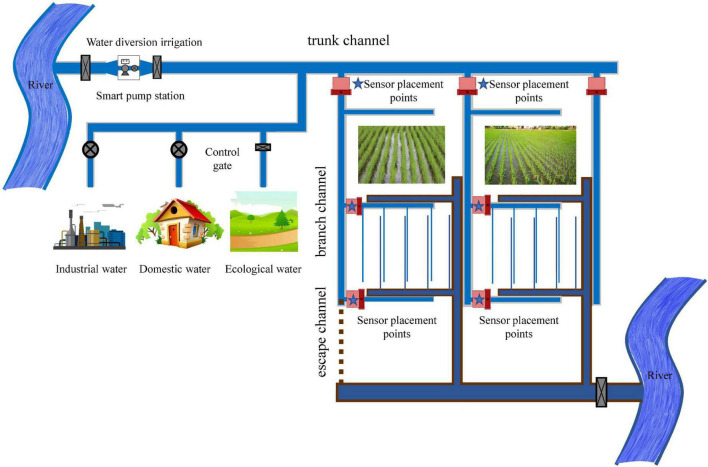
The whole process of water transmission and distribution in the irrigation area and the layout of flow measurement facilities.

Velocity distribution in smooth open channels has been extensively studied. However, many artificial and natural open channels are characterized by vegetation cover. Aquatic vegetation patches are an important part of the river ecosystem. Natural vegetation is an effective measure to protect slopes and soils, purify water quality, and improve the ecological environment of rivers for river ecosystem restoration. It is crucial to study the flow characteristics of rivers with vegetation. The current knowledge of the flow of these rivers is limited to the cross-sectional mean flow velocity and flow depth (Wang et al., 2015, 2019), but the calculation of river flow requires cross-sectional flow distribution. Flow measurement and control facilities are located throughout the irrigation district’s water delivery and distribution, especially at the bifurcation of the final channel system (Figure 1). Devices equipped with sensors are typically used to measure flow in irrigation areas. Flow measurement is inseparable from flow rate measurement. In a closed pipe or gate box, the propagation path depends on the chosen method of placing the sensor in the pipe, and the flow rate can be calculated from the measurement time (Munasinghe and Paul, 2020).

How sensors can be placed to achieve good measurements and reduce workload has always been an area of interest and concern for scientists. Some products have developed an integrated plate gate flow meter that primarily uses Sonaray acoustic array technology. The technology provides an accurate pattern of flow velocity distribution within the metering chamber by mapping flow velocity states through multi-path cross-sections. Thirty-two individual acoustic sensors are placed in four chambers spanning eight planes within the chamber. Flow velocities are sampled in eight planes within the metering chamber, with each plane sampling all flow velocity fields in that plane using acoustic cross-transfer times. The vertical integration of the horizontal flow velocity distribution constitutes a three-dimensional flow velocity distribution. Yakunin (2017) presented a sensor arrangement that which when the sensors are located at the vertices of the tetrahedron, a minimum number of sensors can be used, making it possible to measure the velocity of the air in all three directions simultaneously. Jin et al. (2011) designed a theoretical model of an 18-channel ultrasonic flow sensor based on a time-difference ultrasonic flow meter. Jin et al. (2011) proposed to find the optimal location for placing the sensors based on maximizing the gram-rank equation of the sensor response at each possible location or combination of locations. Oehler and Illingworth (2018) discussed the feasibility of effective feedback control with a single sensor and a single actuator. However, commercial sensors for agriculture and its irrigation systems are very expensive (Garcia et al., 2020). Therefore, how to improve the location of flow sensors, reduce the number of flow sensors and obtain accurate flow estimation is the focus of current research.

A new calculation method is proposed in this article which differs from the previous methods. This simplified method is used to estimate the flow velocity from multiple specific sensing points of flow velocity sensors in an open channel. First, this article is conducted to obtain the flow velocity distribution characteristics of the channel through theoretical analysis. Then, the theoretical expressions for the location of sensors and the formulae for the calculation of the channel flow are given in this article. Finally, based on the results obtained, the flow rate can be quickly obtained by measuring the velocity at these points using the sensors and substituting them into the discharge calculation equation. In addition, relevant experiments were designed involving three cross-sectional types of channels: rectangular, trapezoidal with a curved bottom, and U-shaped. Based on the physical experimental model, it is verified that the above theoretical analysis is reliable in both vegetated and smooth channels.

## Materials and methods

### Location of sensor representative points: Smooth channel as an example

In irrigation areas, the accurate measurement of flow in various channels is an important issue in agricultural engineering. The velocity distribution in smooth open channels has been studied extensively. Log-law was considered to describe the time-averaged flow velocity distribution of a fully developed open channel with uniform turbulent flow, which was first proposed by Keulegan (1938).


(1) $$\begin{array}{c} u^{+}=C\,l\,o\,g\,y^{+}+D \end{array}$$

where *u*^+^ = *u*/$u_{*}$ , *A* = 2.3/κ , $u_{*}$ is shear velocity, *u* is the flow velocity. *C* and *D* are constants and _κ_ is the Karman constant.

Many researchers have demonstrated the validity of this law in different experiments (Kirkgoz, 1989; Tominaga and Nezu, 1991). Nonetheless, experiments have shown that the log-law normally deviates from experimental data in the outer region (Steffler et al., 1985). Coles (1956) proposed adding a wake function to the log-law to represent the mean velocity profile of the boundary layer, which was called the Wake law.


(2) $$\begin{array}{c} \frac{u}{u_{*}}=\frac{1}{\kappa }\,l\,n\,\frac{y}{y_{0}}+\frac{2\,\Pi }{\kappa }\,s\,i\,n^{2}\,\left(\frac{\pi \,y}{2\,h}\right) \end{array}$$

where $u_{*}$ is the bed-shear velocity,κ≈0.41, Π≈0.20≈κ/2 is the wake strength coefficient, and *h* as the flow depth. Herein *y*_0_ is the zero-velocity bed defined by the log-law (first term).

Arc-bottom channels make up the majority of both natural and artificial channels. Arc-bottomed channels, important irrigation and drainage channel, are characterized by high flow rates. Generally speaking, the transfer of flow residual energy takes place along the relatively shortest distance to the boundary (Han et al., 2015). For an arc-bottomed channel, as the channel boundary is curved, we can connect the center of the circle to any point in the channel cross-section and extend it to get to the intersection point with the sidewall. Then, the length between these two points can then be obtained as the relative shortest geometric distance for energy dissipation. The objects of study in this article are arc-shaped channels and rectangular open channels, of which arc-shaped channels include U-shaped channels, and trapezoidal channels with arc bottom. The wall shear force for constant uniform flow in an arc-shaped smooth open channel is the same, and the velocity distribution follows the log-law. Take the trapezoidal and U-shaped channels at the bottom of the arc as examples, the position of the representative point of the mean velocity of the section normal line is deduced.

Based on the zoning theory and the quadratic wake function proposed by Coles (1956), the channel velocity distribution model can be written as the following general expression:


(3) $$\begin{array}{c} \frac{u}{u_{*}}=A\,l\,n\,\left(l\,u_{*}\right)+B\,s\,i\,n^{2}\,\left(\frac{\pi \,l}{2\,K_{l}}\right)+C^{{}^{\prime }} \end{array}$$

where *A*, *B*, and *C’* are correlation coefficients, which are related to the experiment. $u_{*}$ is the local friction velocity. ν is the kinematic viscosity coefficient of water. *l* is the normal distance from each point on the normal line to the side wall. *K*_*l*_ is the vertical distance from the sidewall to the boundary line. The tail flow function part of Eq. (3) is changed according to the trigonometric relationship as follows:


(4) $$\begin{array}{c} \frac{u}{u_{*}}=A\,l\,n\,\left(l\,u_{*}\right)+B\,s\,i\,n^{2}\,\left(\frac{1-c\,o\,s\,\left(\frac{\pi \,l}{K_{l}}\right)}{2}\right)+C^{{}^{\prime }} \end{array}$$

At any point in the channel, take the microelement with length and width of *K*_*l*_ and *dw*, and since *dw* tends to 0, the flow through the microelement can be expressed as:


(5) $$Q=u\cdot K_{l}\cdot d\,w$$

**FIGURE 2:**
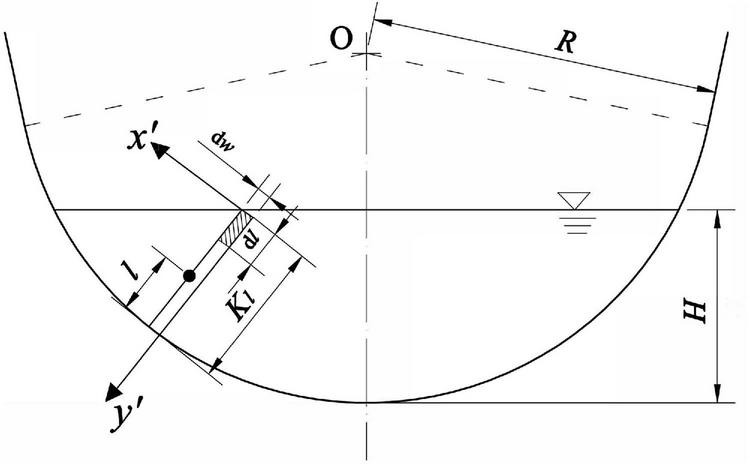
The U-shaped channel is used as an example for analytical derivation. In a U-shaped channel with radius *R* and maximum water depth *H*, take a normal line passing through the center of the circle, and the length under the water surface is *K*_*l*_.

Furthermore, the micro-element rectangle with length and width of d*l* and *dw*, respectively, are taken as shown in the shaded part in Figure 2, so that the flow through the micro-element rectangle is:


(6) d⁢Q=u⋅d⁢w⋅d⁢l

The flow rate is obtained by integrating dl in the *y’* direction:


(7) $$Q=d\,l\cdot \int_{0}^{K_{l}}u_{*}\,\left[A\,l\,n\,\left(l\,u_{*}\right)+B\,\left(\frac{1-c\,o\,s\,\left(\frac{\pi \,l}{K_{l}}\right)}{2}\right)+C^{{}^{\prime }}\right]\,dw$$

At the same time, the flow can be expressed as Eq. (8).


$$Q^{{}^{\prime }}=u\cdot K_{l}\cdot d\,w=K_{l}\cdot d\,w\cdot u_{*}$$

(8) $$\left[A\,l\,n\,\left(l\,u_{*}\right)+B\,\left(\frac{1-c\,o\,s\,\left(\frac{\pi \,l}{K_{l}}\right)}{2}\right)+C^{{}^{\prime }}\right]$$

The flow velocity obtained using integration is equal to the flow rate obtained by the velocity-area method, that is *Q* = *Q*′, the expression of *l* is obtained by simultaneous combining Eqs. (7) and (8), that is:


(9) $$l\,n\,\left(\frac{K_{l}}{e\,l}\right)=K\,c\,o\,s\,\left(\frac{\pi \,l}{K_{l}}\right)$$

(10) $$K=\frac{A}{B}$$

(11) $$t\,s\,i\,n\,t=\frac{2\,A}{B}$$

where $\frac{\pi }{K\,l}\,l=t,t\in \left(0,\pi \right)$. Therefore, the solution of the representative point location needs to be related to the coefficients *A* and *B* in the velocity distribution law, and the values of *A* and *B* are obtained from experiments.

### Simplified flow velocity sensor placement in vegetated channels

In nature, open channels are not completely smooth, that is, flexible vegetation accounts for a large proportion of the channel, so the study of hydraulic characteristics of channels containing flexible vegetation has been widely concerned in recent years. Unlike rigid vegetation, flexible vegetation will bend under the action of water flow, and with the change of flow velocity, the flow velocity will also change due to the influence of flexible vegetation bendings, such as the contact area between vegetation and water flow, and the resistance of vegetation to water flow. Therefore, the study of the effects of submerged flexible vegetation on the physical process of water flow, such as flow structure and flow characteristics, will contribute to the protection and restoration of water ecology. When studying the velocity distribution of vegetated channels, the flow is generally stratified in the vertical direction, which is called the zoning theory. Due to the difference in zoning theories, the velocity distribution models of fully submerged flexible vegetation channels have different expressions and cannot be described by a unified function. Most researchers have obtained the velocity distribution models based on the log-law of velocity distribution in a smooth open channel mentioned above. The following mainly introduces the velocity distribution model based on the two-zone theory. Kouwen et al. (1969) assumed the mixing length and put forward the velocity distribution model based on the classical log-law. The velocity distribution model of the upper part of the plant layer is as follows:


(12) $$\frac{u}{u_{*}}=\frac{u_{h_{v}}}{u_{*}}+\frac{1}{\kappa }\,l\,n\,\left(\frac{y}{h_{v}}\right)$$

where *u*_*h_v_*_ is the time mean velocity of the top of the plant after lodging along the water flow direction and the distance from the channel bottom is *h*_*v*_ . *h*_*v*_ is the average height of plants after bending. κ is Carmen constant. *y* is the distance from any point to the bottom of the channel.

In vegetated channels, generally, the constant, and uniform flow of fully submerged flexible vegetation channels is divided into the vegetation layer and a free water layer in the normal line direction. In the vegetated layer, the resistance per unit of fluid mass caused by vegetation can be described as:


(13) $$F=\frac{1}{2}\,\rho \,C_{d}\,m\,A\,u_{u\,d}^{2}$$

where *A* = *h*_*v*_⋅*d* represents the contact area of each grass with water, _ρ_ is the density of water body, *C*_*d*_ is the drag coefficient of plants, *d* is the diameter of plants, *u*_*ud*_ is the average velocity of the vegetation layer, and *m* is the density of vegetation.

According to Yang and Choi (2010), *u*_*ud*_ can be calculated by the following Equation:


(14) $$u_{u\,d}=\sqrt{\frac{2\,g\,J\,H}{C_{d}\,m\,D\,h_{v}}}$$

In a uniform flow, the shear stress can be expressed as:


(15) $$\tau =\rho \,u_{*}^{2}$$

The shear stress in the upper layer of vegetation is not equal to the resistance per unit of fluid mass caused by the vegetation. Therefore, the average flow velocity of the vegetation layer is:


(16) $$u_{u\,d}=\alpha \,\sqrt{\frac{2\,u_{*}^{2}}{C_{d}\,m\,A}}$$

The coefficient α is introduced to correct the equation. In the free water layer, according to the principle of force balance, the equation of water flow in the non-vegetation layer of submerged vegetation under the condition of constant uniform flow is:


(17) $$\frac{\tau }{\partial y}+\rho \,g\,J=0$$

where _τ_ is stress, ρ is density, and *J* is slope. Ignoring the viscous stress, the stress is equal to Reynolds stress, that is:


(18) $$\tau =-\rho \,\bar{u^{{}^{\prime }}\,w^{{}^{\prime }}}$$

Integrate the above Equation from the water surface down to y in the upper vegetation layer, and the distribution relationship of Reynolds stress can be obtained as follows:


(19) $$-\rho \,\bar{u^{{}^{\prime }}\,w^{{}^{\prime }}}=\rho \,g\,J\,\left(H-h_{v}\right)$$

Introducing Prandtl’s mixing length theory and vegetation riverbed theory (Huai et al., 2009), channel flow velocity distribution formula with flexible vegetation can be written as:


(20) $$\frac{u}{u_{*}}=\sqrt{\frac{H}{L_{n}}}\,\left(\frac{H}{H-h_{v}}\right)\,\frac{1}{\kappa }\,l\,n\,\left(\frac{y}{h_{v}}\right)+\frac{u_{u\,d}}{u_{*}}$$

Considering the reproducibility and uniformity of the experiment, the material of the invertible vegetation used in this study was plastic. The vegetation had a diameter of 3 cm and a height of 6 cm and was fixed to the inner wall of the channel without gaps to simulate the full coverage of vegetation in the channel or river. Eq. (20) reflects the influence of vegetation on the flow velocity of the upper layer from an overall perspective. The flow velocity near the vegetation close to the riverbed approaches zero (Tang et al., 2020). It should also be noted here that although the analysis of the flow inside the vegetation layer is neglected, the flow velocity of the water above is affected by the vegetation layer and is reflected in the flow velocity distribution expression, so it is applicable to the vegetation channel.

In the channel, the water flow scours the vegetation causing it to tilt. We measure the average height of the vegetation after falling as its boundary. In this study, it is considered that the flow velocity in the flexible vegetation layer is constant, i.e., the flow velocity in the vegetation layer is _*u_ud_*_. The flexible vegetation used in this study is densely distributed and low in height, so the flexible vegetation can be analyzed as a whole. The height of the flexible vegetation used in the experiments is low. The focus of this study is on the flow above the vegetation layer. Considering that the inversion height of the vegetation layer in this study is small, resulting in a small flow response in the vegetation layer.

Similar to the derivation process in a smooth channel, based on the channel velocity distribution model with full coverage of flexible vegetation, the position of the representative point of the mean velocity in the free water layer can be obtained:


(21) $$y=\frac{K_{z}}{e}$$

*K*_*z*_ is the vertical distance from the side wall to the boundary line.

## Experimental setup

**FIGURE 3:**
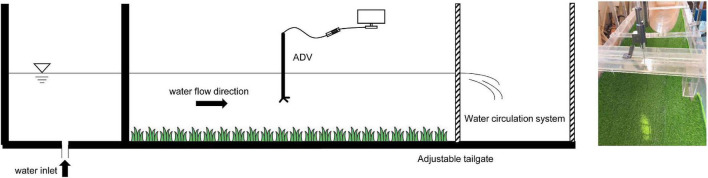
Vegetated channel circulation system device arrangement.

The experiments set vegetated channels and smooth channels. The side wall of the smooth channel is plexiglass, and for smooth channels, flexible vegetation covers the inner wall of the channel (Figure 3). To increase the reliability of the test, there are three types of channel sections in the table: arc-bottom trapezoidal, U-shaped, and rectangle. Tables 1, 2 have illustrated the flow characteristics and parameters for the smooth and vegetated channels.

**TABLE 1:** Flow characteristics and parameters for the smooth channels.

Cross-sectional shape	Arc radius (m)	*Q* (m^3^/h)	*H* (m)	Bottom slope
Arc-bottom trapezium (M1–M3)	0.190	17	0.124	0.067%
		25	0.149	
		27	0.155	
U-shaped (N1–N4)	0.250	30	0.138	0.067%
		40	0.162	
		110	0.263	
		120	0.278	

**TABLE 2:** Flow characteristics and parameters for the vegetated channels.

Cross-sectional shape	Arc radius (m)	*Q* (m^3^/h)	*H* (m)	Bottom slope
U-shaped (T1–T3)	0.250	40	0.185	0.067%
		50	0.214	
		60	0.248	
Rectangle (H1–H3)	–	87.84	0.298	0.035–0.045%
		88.20	0.264	
		91.80	0.275	

The experiments are carried out in the Hydraulic Experiment Hall of China Agricultural University (CAU) (Beijing, China) and the Hydraulics Laboratory at the Wuhan University (WHU) (Wuhan, China) (Huai et al., 2009). The main components of the channel are the head tank, the tail tank, the glass channel, and the water circulation system. The water tank is aligned with the center of the channel and symmetrical with the centerline of the glass channel. Also, additional honeycomb steel plates are installed at the entrance of the channel for a more uniform flow rate. An adjustable tailgate was installed at the end of the channel to vary the water depth. The cross-section of the smooth arc-bottomed channel is composed of an arc section at the bottom and a straight section connected with an arc section at both ends, its bottom arc is 0.19 m radius and 90° right angle arc, the side slope coefficient of a straight section at both ends is 1:1, the height of the channel bottom from the endpoint of the straight section is 0.18 m. The channel is fixed by using a steel structure bracket. The cross-section of the U-shaped vegetated channel consists of a circular arc at the bottom and a straight section at the top, and the flexible vegetation fully covers the inner wall of the channel. The total length of the test channel section is 6.7 m, the radius of the bottom arc of the flume is 0.25 m, the flume is fixed on the steel structure support, and the bottom slope of the channel chosen for this test is 1:1,500. An adjustable tailgate was installed at the end of the channel to vary the water depth. Considering the reproducibility and uniformity of the experiment, the material of the invertible vegetation used in this study was plastic. The vegetation had a diameter of 3 cm and a height of 6 cm and was fixed to the inner wall of the channel without gaps to simulate the full coverage of vegetation in the channel or river.

To ensure the relative accuracy of flow velocity measurement, the location of flow velocity measurement in the test is chosen at 2/3 of the channel length. Due to the symmetry of the channel section, the velocity distribution of the section should also be symmetrical, so the test only needs to measure the test data in half of the area with the mid-pipeline as the dividing line. In this test, a measurement line is arranged every 2 cm from the mid-pipeline to the right, and a measurement point is arranged every 1 cm for each measurement line to ensure that the measured data can accurately reflect the actual flow field.

The test needs to ensure that the water flow in the channel is uniform in the open channel, therefore, the water supply flow needs to be adjusted to keep the channel flow uniform and stable. The flow rate in the test is determined using a flow meter. Common current flow velocity measurement sensors are Acoustic Doppler Velocimetry (ADV) and propellers, both of which are used in the experiments. Flow velocity measurements are performed by ADV at measurement points on the channel, each of which is a grid node divided in advance, and the measured instantaneous flow velocity data can be transferred directly to a computer. The axes of the experiment are set as follows: x-axis for the longitudinal direction, z-axis for the lateral direction, and y-axis for the vertical direction. To study the variation of water flow characteristics of submerged vegetation along the water depth direction, the experimental measurements were carried out in the XY plane. To obtain the flow velocity data by using ADV, which needs to be installed on a rail located above the water tank that can move in both lateral and longitudinal directions. Normally observation is made at 0.1 depth increments between 0.1 and 0.9 of the total water depth. The minimum depth of the instrument is 2 cm. In the experiment, we ensure that the distance between the water surface and the instrument, and between the bottom of the canal and the instrument, is within the instrument specifications. In order to minimize the influence of ADV noise, the sample size of each measurement point in this experiment is about 2,000 times, 120 s of data are collected at each test point with a sampling frequency of 25 Hz, after which the computer software automatically generates an Excel containing the data in the specified folder, and finally, the acquired instantaneous velocity is averaged.

## Results

### Cross-sectional flow velocity distribution based on test data

**FIGURE 4:**
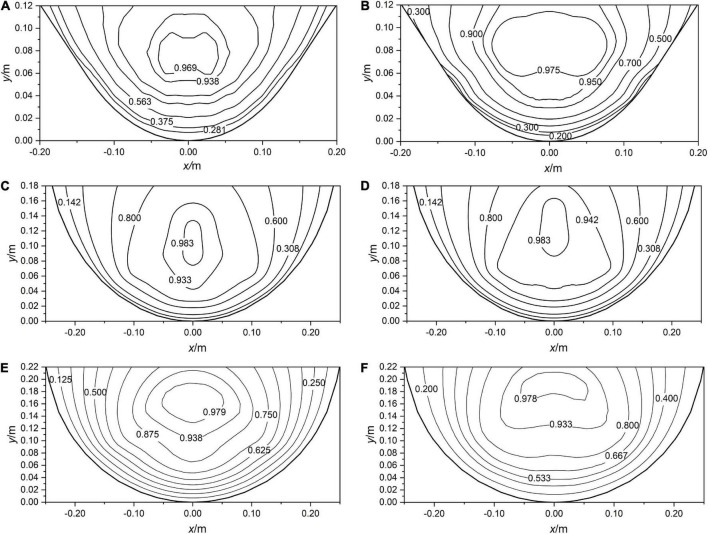
The contour distribution of velocity normalized in channels under M1 **(A)**, M2 **(B)**, N1 **(C)**, N2 **(D)**, T1 **(E)**, T2 **(F)**.

According to the experiment results, the contour distribution of channel section velocity can be expressed. As can be seen from Figure 4, the magnitude of flow velocity in the cross-section is symmetrically distributed along the midline, and the core area of flow velocity is located below the water surface. The flow velocity in the vegetation layer is smaller, which indicates that the resistance is higher in the flexible vegetation layer. The flow velocity distribution pattern of water above the vegetation layer is similar to that of the smooth open channel, and all of them show a dip-phenomenon near the water surface. In addition, the empirical equation of the flow distribution shows that the inverted height *h*_*v*_ and *u*_*ud*_ in the vegetated channel also affect the flow distribution. This is the reason why the contours of flow velocity distribution in the vegetated channel do not appear to show very regularly.

### Velocity distribution along normal line direction in channels

**FIGURE 5:**
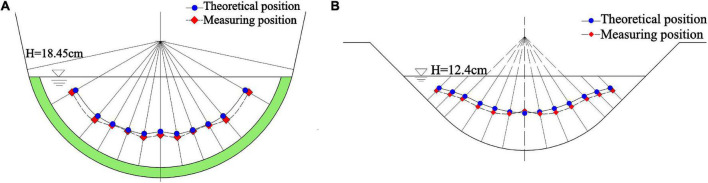
Comparison between theoretical position and measured position of representative point of normal average velocity. **(A)** vegetated U-shaped channel, **(B)** smooth arc-bottom trapezium channel.

In section “Materials and methods,” there are two representative points of the mean velocity derived along a normal line of smooth channel boundary and only one point in the vegetated channel yet. Since the representative point near the water surface is susceptible to surface turbulence and secondary flow, the representative point near the bottom of the channel is chosen for the following analysis. The normal measurement line is used as the study object, and the location of the representative mean flow velocity is plotted on each normal line. The theoretical location value is the location of the representative point of the mean flow velocity obtained from the above mechanism analysis, and the measured value is the location obtained by interpolation after averaging the measured flow velocity data. The results are shown in Figure 5.

**FIGURE 6:**
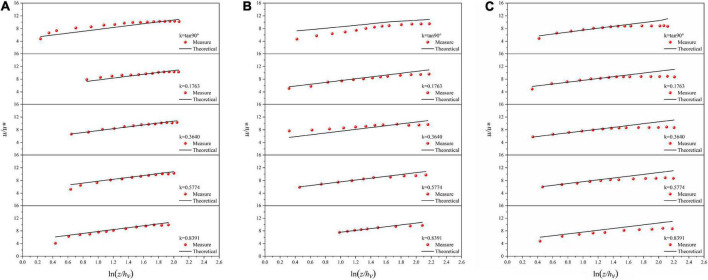
Velocity distribution along normal line directions in vegetated channel under T1 **(A)**, T2 **(B),** and T3 **(C)**.

Unlike the smooth channel, in the vegetated channel, *H*, *L*_*n*_, and *h*_*v*_ are different for different flows, especially *L*_*n*_ in the normal direction of the sidewall is affected by both the water depth and the normal position, so the coefficients before different log expressions change with these parameters. So, the exact parameters are not given for the vegetated channels. In a vegetated channel the velocity distribution data of five normal line directions under three flow rates are expressed in the form of *u*/$u_{*}$ and *ln*⁡(*z*/*h*_*v*_), and the theoretical values are calculated by Eq. (20). Figure 6 are as follows:

For smooth channels, first of all, the velocity data on the middle perpendicular are fitted by the empirical formula. It is found that the fitting formula is consistent with Eq. (3). Popularize and verify the formula to check whether the measured velocity data of the whole cross-section are in accordance with the formula. According to the fitting of three groups of test results, the values of *A* and *B* can be determined:


(22) $$\left\{ \begin{array}{c} A=2.5\\ B=\frac{1.3}{0.41} \end{array}\right.$$

**FIGURE 7:**
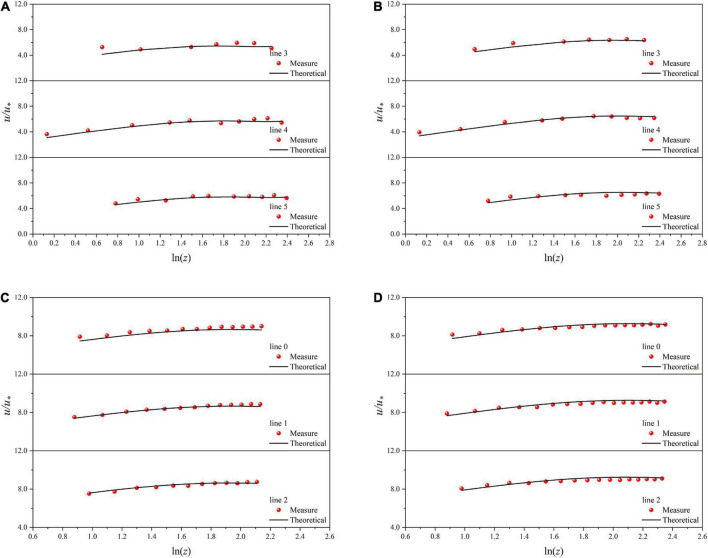
Velocity distribution along normal line directions in smooth channel. **(A)** M1, **(B)** M2, **(C)** N1, **(D)** N2.

This result is very close to the relevant parameters in the classical log-law. Moreover, many researchers have given values of the parameters for specific working conditions, and their magnitudes are all around these two values. Therefore, in this test, the values of the two parameters A and B are reliable after the verification of a large amount of data. The velocity distribution data are expressed in the form of *u*/u* and *ln*⁡(*z*), and the theoretical values are calculated by Eq. (3). The results are shown in Figure 7.

## Discussion

### Analysis of flow velocity along normal line directions

**FIGURE 8:**
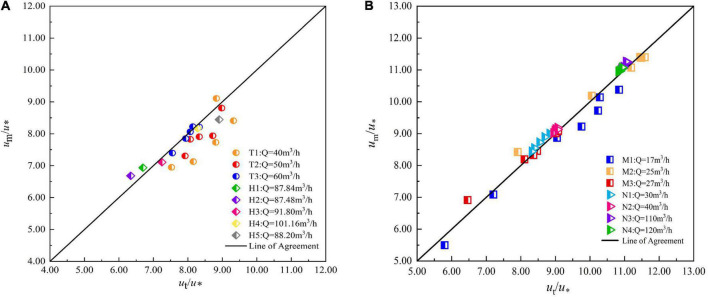
Comparison between the experimental and theoretical values of the mean velocity in the arc-bottom trapezoidal channel. **(A)** vegetated channel, **(B)** smooth channel.

The measured flow velocity at the representative point of normal average flow velocity is taken as theoretical value *u*_*t*_, and the flow velocity obtained by averaging the flow velocities at all normal measuring points is taken as experimental value *u*_*m*_, which is divided by $u_{*}$ to be dimensionless. The theoretical value (*u*_*t*_/$u_{*}$)of the representative point is taken as abscissa and the experimental value (*u*_*m*_/$u_{*}$) is taken as ordinate and plotted in the coordinate system. It can be seen from Figure 8 that the flow velocity data of all working conditions are distributed near the straight line with slope 1.

In a vegetated channel, as the flow rate increases, the flow is closer to uniform flow, which makes the difference between the measured and theoretical values decrease. In all three flow cases, the flow velocity at the measurement points away from the sidewall on each normal line gradually deviates from the theoretical value. It shows that the log-law cannot explain the flow velocity distribution law near the water surface well, and the tail flow function needs to be added for correction.

In a smooth channel, the calculation revealed that in our test, from the mid-pipeline to close to the water surface, the normal error situation there is a trend of first decreasing and then increasing, indicating that in the channel quarter of the place, the measured value and equation can be a good match, and overall, the measured and calculated values of flow velocity are relatively close, the relative error is within 10%.

Compared with the four normal lines on the side (L1, 2, 3, and 4), the flow velocity distribution at the mid-pipeline (L0) deviates more from the theoretical value, which may be caused by the following two reasons:

I. In the case of flexible vegetation in the channel, the flow measurement point at the centerline of the cross-section is far from the full grass sidewall on both sides, which is less affected by the flexible vegetation and the flow velocity distribution is closer to the smooth open channel condition.
II. Secondary flow may exist in the open channel flow section.

### A simplified algorithm for discharge estimation

Since the equations for the location of characteristic sensing points of the flow velocity sensors were obtained in section “Materials and methods,” we can estimate the discharge with multiple characteristic sensing points’ velocities by setting representative measurement lines. Therefore, the vertical line and two other normal lines of the channel as the measurement normal lines were selected for the distribution and control of measurement points.

**FIGURE 9:**
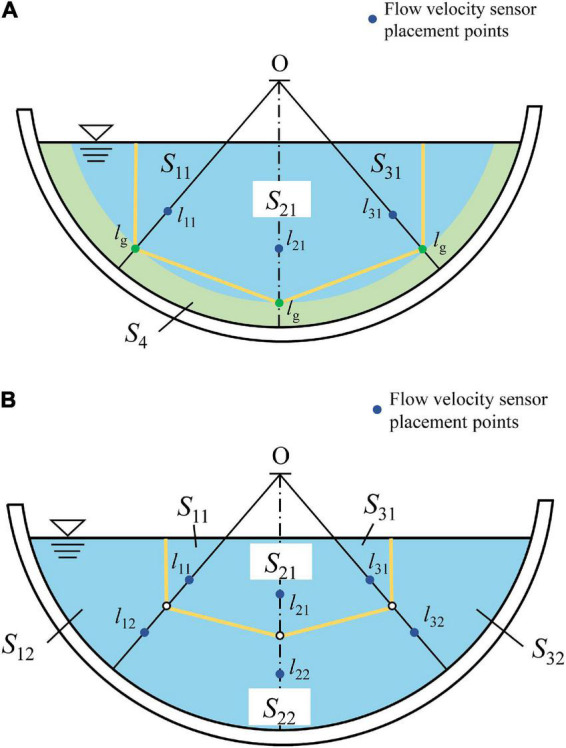
Diagram of estimating discharge with 3-measuring-lines. The positions of *l*_11_, *l*_12_ … *l*_32_ are determined by Eqs. (11) and (21), and S_11_, S_21_… S_4_ are the areas of the polygon. **(A)** vegetated channel, **(B)** smooth channel.

If the channel does not contain vegetation, firstly, the locations of two normal average velocity representative points are determined on each of the three normal lines. Next, find the average position of the two points on the normal. Use the averaged point as the point for zoning and flow calculation. Connect the three partition points. The points on the two inclined normal lines intersect vertically with the water surface to form areas S11, S21, S31, and S4. The middle part of the channel is divided into areas S21 and S22 in smooth channel. The total flow rate of the channel can be calculated in zones, and the flow rate of each zone can be obtained by multiplying the flow rate measured by the flow sensor by the corresponding area. Nevertheless, for channels containing vegetation, in addition to considering the three areas, we can also obtain the flow of the vegetation layer by multiplying the top flow rate of the vegetation layer with the area of the vegetation layer, which is obtained by placing a flow sensor above the vegetation layer (Figure 9).

Considering that there are two channels with different roughness, including smooth and flexible vegetated channels, according to the velocity-area method, there are two formulas for calculating the total flow of channels:

(1) In the smooth channel, there are three partition polygons divided by the above method, and the total *Q* is:


(23) $$Q=\alpha \,u_{1}\,\left(S_{11}+S_{12}+S_{31}+S_{32}\right)+\beta \,u_{2}\,\left(S_{21}+S_{22}\right)$$

(2) In the flexible vegetated channel, there are four partition polygons divided by the above method, and the total flow *Q* is:


(24) $$Q=\alpha \,u_{1}\,\left(S_{11}+S_{31}\right)+\beta \,u_{2}\,S_{21}+\theta \,u_{g}\,S_{4}$$

where *u*_1_ is the velocity at the flow sensor placement point on the two inclined normal lines and *u*_2_ is the velocity at the flow sensor placement point on the mid-slope normal. α, β and θ are the flow correction factors, determined from the test data.

The two measurement normal lines at different locations affect the accuracy of the flow calculation. We use M1, M2, and M3 conditions as examples (Table 3). The normal line 3, located at about a quarter of the channel, is used to calculate the results of the flow with the smallest average error. Therefore, considering the average error and operability, as well as simplicity in practice, we conclude that the choice of the normal located at about a quarter of the channel is most suitable for measuring the discharge of the open channel.

**TABLE 3:** Comparison of errors in applying two different normal lines to calculate flow rates.

Conditions Measuring lines	M1	M2	M3	Average error
Normal line 1	−19.21%	−3.73%	−3.32%	−8.75%
Normal line 2	−14.09%	−38.00%	−41.29%	−31.12%
Normal line 3	−5.52%	5.08%	1.24%	3.94%
Normal line 4	−4.23%	12.44%	13.43%	7.22%
Normal line 5	−0.67%	12.37%	14.34%	8.68%
Normal line 6	4.65%	12.28%	16.00%	10.98%

**FIGURE 10:**
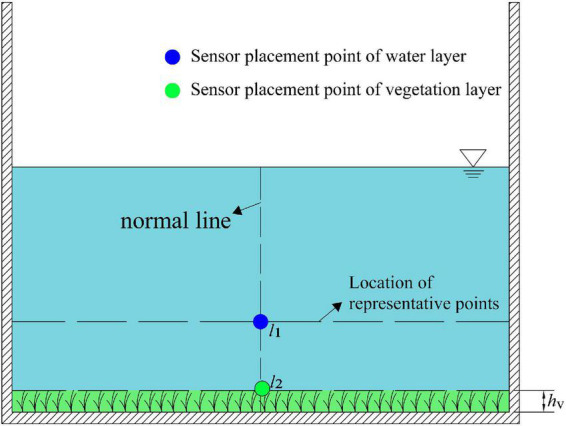
The schematic diagram for estimating the discharge of a rectangular vegetation-containing channel using one measurement line. The position of *l*_1_ is determined by Eq. (21), and the position of *l*_2_ is the height of the vegetation canopy after vegetation fall, measured from the experiment. *S*_1_ is the area of the water layer and *S*_2_ is the area of the vegetation layer.

For the rectangular vegetation-containing channels, the normal lines of the bottom walls are all perpendicular to the walls, so the positions of the representative points on the normal lines are located at the same height. As shown above, the representative point on the mid-pipeline is selected to place a flow sensor. The measured flow rate is used as the average flow rate of the upper water layer, and the flow sensor is selected to place a flow sensor at the height of vegetation inversion on the mid-pipeline (Figure 10). The measured flow rate is used as the average flow rate of the vegetation layer, so the section flow rate calculation formula can be expressed as Eq. (25).


(25) $$Q=\alpha \,u_{1}\,S_{1}+\beta \,u_{2}\,S_{2}$$

The calculation formula of the average error value (*e*) in Table 4 is given. The accuracy of the flow calculation method is high, and its relative errors can all be controlled at about 5%, indicating that our proposed flow measurement calculation formula is feasible and can achieve the purpose of simplifying the flow measurement method and improving the accuracy of flow measurement.


(26) $$e=\frac{Q_{t}-Q_{m}}{Q_{m}}$$

**TABLE 4:** Relative error in calculating the flow rate.

Experiment conditions	Observed value *Q*_*m*_/(m^3^/h)	Calculated value *Q*_*t*_/(m^3^/h)	*e*
M1–M3	17	16.06	−5.52%
	25	26.27	5.08%
	27	27.34	1.24%
N1–N3	30	27.32	−8.94%
	40	39.21	−1.98%
	110	117.20	6.55%
	120	124.86	4.05%
T1–T3	40	42.31	5.78%
	50	51.53	3.07%
	60	63.74	6.24%
H1–H3	87.84	85.84	−2.28%
	88.20	93.65	6.07%
	91.80	82.84	9.75%

The results of the calculation of the relative error are shown in Table 4. The actual flow rate of the channel is the flow rate indicated by the test electromagnetic flow meter, and the theoretically calculated flow rate is the result obtained from the calculation formula.

However, in fact, this simplified method of estimating the flow velocity does have some deficiencies.

(1) For the channels studied in this article, there are only a few different cases of channel-related parameters, such as the degree of the bottom arc of the channel and the height of the vegetation in the channel. More laboratory tests and field experiments can be added in the future to explore whether the conclusions are also applicable.
(2) In the verification of the measurement point layout and flow measurement method, this article also needs to determine flow correction for different tests. However, questions such as how the correction should be made and whether there is a general rule for the correction coefficients need to be further improved by subsequent experiments and theoretical analysis.

## Conclusion

Vegetated and smooth open channel flows are revisited in terms of the cross-sectional velocity distributions and flow measurement. Through mechanistic analysis and physical experiments, a new method for estimating channel flow by flow velocity sensors is proposed with the following main conclusions:

(1) In ecological channels, representative points indicating the average flow velocity exist in the normal line direction. In practice, the average flow rate of each measurement line can be quickly obtained by using flow velocity sensors for flow rate measurement. Especially for ecological channels, the influence of vegetation on the measurement can be reduced without affecting its accuracy. A simplified sensor flow measurement method facilitates the construction and restoration of ecological channels.
(2) The characteristics of the flow velocity distribution along the normal line directions in an ecological channel are obtained by theoretical analysis, and the expression of the multiple characteristic sensing points locations of the flow velocity sensor in the channel section is proposed. The velocity deduction based on log-law indicates the average velocity in the normal line direction under different flow rates and roughness, which fits perfectly with the experimental data.
(3) Based on the theory of representative points, the formula of ecological channel flow is given. By comparing the effects of applying the normal lines at different locations on the flow calculation results, we find that the normal lines located at the quarter of the ecological channel have the smallest relative errors. The validity of the flow calculation formula is proved by numerous experimental data.

## Data availability statement

The raw data supporting the conclusions of this article will be made available by the authors, without undue reservation.

## Author contributions

YZ: conceptualization, methodology, formal analysis, investigation, resources, data curation, writing—original draft, and visualization. WY and XH: investigation and resources. SW: conceptualization, formal analysis, and validation. TL: writing—review and editing and supervision. YH: conceptualization, validation, writing—review and editing, supervision, and funding acquisition. All authors contributed to the article and approved the submitted version.
